# Characterization of complete mitochondrial genome of *Nemania diffusa* (Xylariaceae, Xylariales) and its phylogenetic analysis

**DOI:** 10.1080/23802359.2019.1704665

**Published:** 2020-01-08

**Authors:** Dexiang Tang, Guodong Zhang, Yao Wang, Mingxi Zhang, Yuanbing Wang, Hong Yu

**Affiliations:** a Yunnan Herbal Laboratory, School of Life Sciences, Yunnan University, Kunming, China;; b The International Joint Research Center for Sustainable Utilization of Cordyceps Bioresources in China and Southeast Asia, Yunnan University, Kunming, China;; c The Research Center of Cordyceps Development and Utilization of Kunming, Yunnan Herbal Biotech Co. Ltd, Kunming, China

**Keywords:** Mitochondrial genome, *Nemania diffusa*, phylogenetic analysis

## Abstract

*Nemania diffusa* is a parasitic fungus of many plant species and causes large economic losses to the forestry industry. In the present study, the complete mitochondrial genome of *N. diffusa* is first reported. The circular genome is 258,879 bp in length, containing 14 protein coding genes, 2 ribosomal RNA genes, 25 transfer RNA genes. The 258,879 bp long mtDNA of *N. diffusa* represents one of the largest sequenced fungal mitogenomes. The overall base composition is 34.4% A, 35.9% T, 14.0% G, 15.7% C and the content of GC is 29.7%. Phylogenetic analysis based on concatenated protein coding genes from 24 species in 8 orders was conducted using Bayesian inference (BI) method. *Nemania diffusa* is clustered in the order Xylariales and is more closely related to *Annulohypoxylon stygium* of Hypoxylaceae. This work facilitates the future study of molecular biology and evolution of xylariaceous fungi.

*Nemania*, belonging to the family Xylariaceae (Xylariales, Sordariomycetes), was established in 1821 for heterogeneous taxa and later the plurivorous genus accommodated numerous species of *Hypoxylon* (Miller 1961). *Nemania diffusa* is synonymous with *Hypoxylon vestitum* and is a parasitic fungus of over 19 plant species such as beech and tea, which causes large economic losses to the forestry industry (Balasuriya and Adikaram 2009; Okane et al. 2012). *Nemania diffusa* produces a series of enzymes such as phenoloxidases, peroxidase, acid phosphatase and laccase, which are able to neutralize fungitoxic compounds like phenols, ferulic acid, tannic acid, etc. (Balasuriya 1998; Rayner and Boddy 1988; Balasuriya and Adikaram 2009). However, the generic concept of *Nemania* has not been clearly confirmed and thus phylogenetic study of the important fungus *N. diffusa* is weak. In this study, we first report the complete mitogenome of *N. diffusa* and determine its systematic position in the order Xylariales, so that it facilitates the future investigation of molecular biology and evolution of xylariaceous fungi.

*Nemania diffusa* strain YFCC 6900 used in this study was isolated from a stroma of natural *Ophiocordyceps highlandensis* collected in Yunnan, China (25°23′16″N, 102°53′42″E, alt. 2697 m). The strain was deposited at the Yunnan Fungal Culture Collection (YFCC), Yunnan University. To extract genomic DNA, mycelia on potato dextrose agar (PDA) plates at 20 °C for 2 weeks were prepared. The total genomic DNA of *N. diffusa* was extracted using MiniBEST Plant Genomic DNA Extraction Kit (TaKaRa, China) following the manufacturer’s protocol. The DNA integrity and quality were verified by electrophoresis on 1.0% agarose gels and the purified DNA was sequenced with the Illumina sequencing platform (HiSeq PE150). Based on the high-quality reads, the complete mitogenome of *N. diffusa* was assembled by SPAdes 3.9.0 with default parameter (Bankevich et al. 2012). The mitogenome was annotated by using MFannot tool and ARWEN web server, combined with artificial correction technology. The Organellar Genome DRAW tool was used to drew the mitogenomic circular map (Lohse et al. 2007).

The complete mitogenome sequence of *N. diffusa* was submitted to GenBank database under accession number no. MN 780510. Its mitogenome is 258,879 bp in length, containing 14 protein coding genes (7 nad genes, 2 atp genes, 3 cox genes, 1 cob and 1 rps genes), 2 ribosomal RNA (1 rns and 1 rnl) genes and 25 transfer RNA (tRNA) genes. The overall base composition is 34.4% A, 35.9% T, 14.0% G, 15.7% C, with a GC content of 29.7%.

Mitogenomic sequences of 23 species in Ascomycota downloaded from NCBI were used to determine the systematic position of *N. diffusa*. The 14 protein coding genes were selected for the alignment by using MUSCLE (Edgar 2004). Bayesian inference (BI) method with the software MrBayes v.3.1.2 was used to construct the phylogenetic tree (Ronquist and Huelsenbeck 2003). The BI analysis was run on the MrBayes v.3.1.2 for 5 million generations using the GTR + G + I model. The phylogenetic tree is composed of 8 orders, viz. Glomerellales, Helotiales, Hypocreales, Microascales, Ophiostomatales, Sordariales, Saccharomycetales and Xylariales (Figure 1). Phylogenetically, *N. diffusa* is clustered in the order Xylariales and is more closely related to *Annulohypoxylon stygium* of Hypoxylaceae.

**Figure 1.**
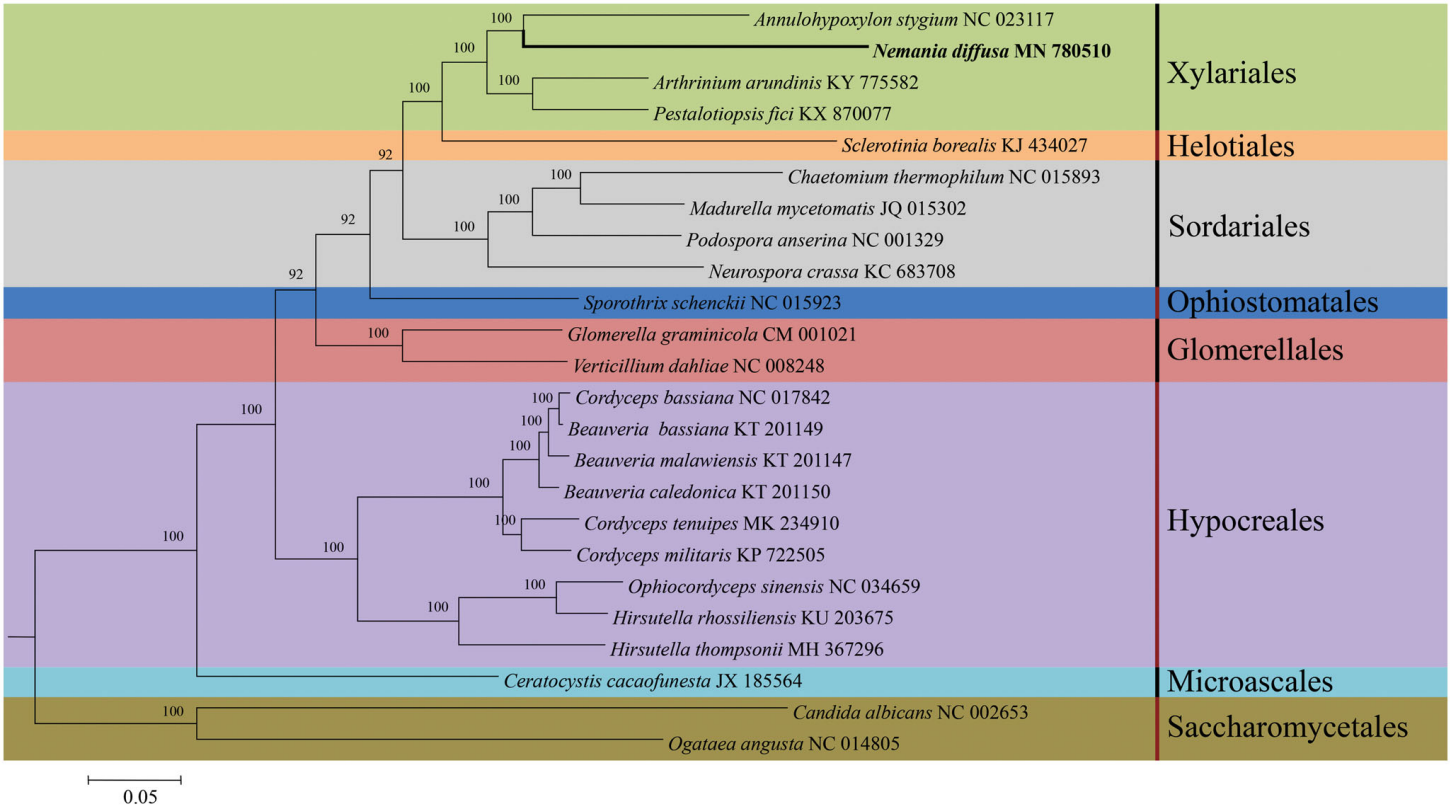
Phylogenetic relationships among 24 species based on Bayesian inference analysis from 14 concatenated mitochondrial protein-coding genes (PCGs). The 14 PCGs include subunits of the respiratory chain complexes (*cob, cox2, cox1, cox3, rps3*), ATPase subunits *(atp6, atp9*), NADH: quinone reductase subunits (*nad4L, nad1, nad2, nad3, nad4, nad5, nad6*).
